# RELATIONSHIP BETWEEN CARE CHALLENGE TYPE AND PSYCHOSOCIAL OUTCOMES IN FAMILY CAREGIVERS OF PERSONS WITH DEMENTIA

**DOI:** 10.1093/geroni/igz038.3340

**Published:** 2019-11-08

**Authors:** Clarissa Shaw, Kristine N Williams, Maria Hein, Carissa Coleman, Yelena Perkhounkova

**Affiliations:** 1 University of Iowa College of Nursing, Iowa City, Iowa, United States; 2 University of Kansas Medical Center, Kansas City, Kansas, United States; 3 The University of Iowa, Iowa City, Iowa, United States

## Abstract

Enhancing dementia care is a public health priority and supporting family caregivers of persons living with dementia (PLWD) is a critical need. This poster reports the relationships between the types of care challenges reported by family caregivers and their scores on psychosocial measures. Family caregivers (N=83) participating in the FamTechCare clinical trial identified three top priority care challenges and completed a series of measures (i.e., burden, depression, sleep quality, and reaction to dementia behaviors) at baseline. Priority care challenges were classified using the 10-category Technology-supported Dementia Care Typology. Three of the categories (i.e., behavioral and psychological symptoms of dementia [BPSD], activities of daily living [ADL], and disease expectations [DE]) were reported by an adequate number of caregivers in order to test relationships with psychosocial measures using the Kruskal-Wallis Test. Caregivers reporting 2 or 3 BPSD challenges had higher burden (p=.007), more depression (p=.022) and worse sleep quality (p=.020) compared to those reporting 0 or 1 care challenges related to BPSD. In comparison, caregivers with 2 or 3 challenges related to DE (e.g., PLWD memory loss) had less burden (p=.008), less depression (p=.030), and better sleep quality (p=.042), compared to those reporting 0 or 1 challenge related to DE. Caregivers identifying 2 or 3 care challenges related to ADLs also reported higher levels of depression (p=.036). Dementia caregivers face vast caregiving responsibilities. Caregivers facing BPSD challenges report greater burden and depression. These results reinforce the need for tailored interventions to assist family caregivers in the managing varied care challenges.

